# No Bilingual Benefits Despite Relations Between Language Switching and Task Switching

**DOI:** 10.3389/fpsyg.2020.01832

**Published:** 2020-07-24

**Authors:** Mona Timmermeister, Paul Leseman, Frank Wijnen, Elma Blom

**Affiliations:** ^1^Department of Special Education, Utrecht University, Utrecht, Netherlands; ^2^Department of Languages, Literature and Communication, Utrecht University, Utrecht, Netherlands; ^3^The Arctic University of Norway UiT, Tromsø, Norway

**Keywords:** child bilingualism, cognitive control, language switching, task switching, executive functions, migrant children

## Abstract

Previous research has shown that bilingual children outperform monolinguals on tasks testing cognitive control. Bilinguals’ enhanced cognitive control is thought to be caused by the necessity to exert more language control in bilingual compared to monolingual settings. Surprisingly, between-group research of cognitive effects of bilingualism is hardly ever combined with within-group research that investigates relationships between language control and cognitive control. The present study compared 27 monolingual Dutch and 27 bilingual Turkish-Dutch children matched on age and fluid intelligence on their performance in a nonverbal switching task. Within the group of bilinguals, the relationship between nonverbal switching and language switching was examined. The results revealed no between-group differences on nonverbal switching. Within the bilingual sample, response times in the language switching and nonverbal switching tasks were related, although no relationships were found between accuracy, switching cost and mixing cost on both tasks. The results support the hypothesis that children utilize domain-general cognitive control in language switching, but this relationship does not entail that bilinguals have better cognitive control than monolinguals.

## Introduction

An important aspect of growing up bilingually is learning to control one’s languages. For example, some bilingual children grow up in single-language contexts where one language is used in one environment and the other language in another environment, as is the case for children who grow up in families where the home language differs from the language used at school. At home, these children need to suppress the language used at school and at school they need to suppress the home language. Other children grow up in dual-language contexts in which both languages are used in the same environments, but typically with different speakers (Green and Abutalebi, 2013), as in bilingual families characterized by a one-parent-one-language pattern. In such a situation, children suppress one of their languages while interacting with one parent and suppress their other language when they interact with their other parent (Verhagen et al., 2017). Both single- and dual-language contexts are common (de Houwer, 2007) and exemplify that bilingual children often need to inhibit one of their languages and resist interference from this language.

Theoretical accounts of bilingual language use, e.g., the Inhibitory Control Model (Green, 1998) or the Adaptive Control Hypothesis (Green and Abutalebi, 2013), suggest that the mechanisms underlying bilingual language control draw on domain-general cognitive control processes, which are also used when switching between different nonverbal cognitive tasks. Because bilingual speakers engage their cognitive control processes frequently to control their language use, the cognitive control processes of bilinguals may be optimized (Bialystok and Craik, 2010; Stocco et al., 2014), leading to cognitive control benefits for bilinguals. In the last decades, the hypothesis that bilingual children outperform their monolingual peers on cognitive control has been explored extensively by comparing bilingual and monolingual children on tasks that test specific cognitive control functions such as attention, switching, and working memory. Many of these studies confirmed the hypothesis that the bilingual children have cognitive control advantages (e.g., Martin-Rhee and Bialystok, 2008; Barac and Bialystok, 2012; Morales et al., 2013; for review studies, see Adesope et al., 2010; Barac and Bialystok, 2011; Hilchey and Klein, 2011). The results of individual studies are not unanimous, however, as there are also studies in which no differences were observed (e.g., Morton and Harper, 2007; Paap and Greenberg, 2013; Duñabeitia et al., 2014; Paap et al., 2015). It has been argued that bilingual effects on executive functions are more prominent in children and elderly people (Bialystok, 2015), although there are also studies that do not find such effects for these age groups (Duñabeitia et al., 2014; Lehtonen et al., 2018). The growing number of studies with null results in this field has created doubts regarding the robustness of effects showing bilingual advantages on executive functions and suggests that effects might depend on specific aspects of the bilingual experience, for example the frequency of language switching in real life (Verreyt et al., 2016; Barbu et al., 2018).

Studies on the relationship between cognitive control and language control within bilinguals and studies that compare cognitive control across bilinguals and monolinguals are typically part of two separate lines of research. The main goal of this study is to combine these lines of research and conduct both a between- and within-group study. Combining the two types of studies is particularly important in light of the variable findings regarding cognitive effects of bilingualism. What we wanted to know, was: Does the presence of a bilingual advantage in a group of bilinguals go hand-in-hand with the expected cross-domain link between language control and cognitive control in the same group of bilinguals and, vice versa, does the absence of a bilingual advantage coincide with the absence of a cross-domain relation? Finding a difference in cognitive tasks between bilinguals and monolinguals without a relationship between language control and cognitive control within the group of bilinguals could suggest that other variables, such as demographic differences (e.g., SES) or task-specific effects are responsible for a bilingual advantage (Paap et al., 2015). Failing to find a between-group difference in the presence of a significant within-group relation demonstrates that the absence of cognitive effects does not necessarily imply the absence of cross-domain links and may suggest that any training effects in the bilinguals are masked by other variables. To investigate these different scenarios, the current study investigated the cognitive switching function. Switching between languages has been found to be effortful (Kohnert et al., 1999) which can be a basis for practice effects (Morton and Harper, 2007) that, in turn, lead to cognitive effects in nonverbal switching.

One type of switching task that has been used repeatedly to study effects of bilingualism on cognitive abilities is a color/shape switching task (e.g., Prior and MacWhinney, 2010; Stasenko et al., 2017), a paradigm in which participants have to identify either the color or the shape of an object presented on a computer screen depending on which rule is cued to be active. After completing single-task blocks in which participants have to respond either *only* to the color or *only* to the shape of an object, they engage in a task switching block. In that block, for each trial the relevant aspect (color or shape) is indicated by a cue and participants have to switch between trials in which they respond to the color and trials in which they respond to the shape of an object. As switching between languages can be regarded as a specific kind of switching, this task appears to be of relevance to bilingual language use, tapping into the domain-general mechanisms that have been claimed to underlie language switching.

Effects of bilingualism on nonverbal switching can be determined by looking at two different dependent variables, which are thought to represent different types of cognitive control, namely switching and mixing cost (Braver et al., 2003). A cued switching test provides not only information about accuracy and response times of switching between different tasks but also allows for calculating different processing costs related to task-switching. The difference in response times between trials where the task changes from responding to color to responding to shape, or vice versa (“switch trials”) and trials where there is no change of task (“repeat trials”) is called *switching cost*. The difference in response times between repeat trials in a switching block and trials in a single task block (*only* respond to color or *only* respond to shape) is called *mixing cost.* It has been suggested that switching costs draw on reactive control processes (Braver et al., 2003), used for stimulus-driven goal reactivation and interference resolution (Braver, 2012), whereas mixing costs may reflect proactive control processes (Braver et al., 2003), where sustained attention is used to maintain goal-relevant information.

A recent review article by Paap et al. (2016) focuses on comparisons between bilingual and monolingual groups on such switching tests and shows that although some studies have reported a bilingual advantage on nonverbal task switching (Prior and MacWhinney, 2010; Prior and Gollan, 2011; Barac and Bialystok, 2012; Wiseheart et al., 2014), other studies yield no significant differences (Tare and Linck, 2011; Prior and Gollan, 2013; Paap and Sawi, 2014; de Bruin et al., 2015). These contrasting findings could be related to a more general issue of studies that make use of a between-group design that compares bilinguals and monolinguals, namely, the difficulty of finding purely monolingual controls (Paap et al., 2016). This issue should be less of a problem when comparing bilingual and monolingual groups of children instead of adolescents or adults, as children who are raised monolingually are often not yet systematically exposed to a second language (L2) during the first years of elementary school. Another potential advantage of studying effects of bilingualism on cognitive control in children, as compared to adults, is that they are still in the early stages of their cognitive development (Anderson, 2002; Carlson, 2005) and therefore are more likely to show variability in cognitive skills than for example young adults who are at the peak of their cognitive abilities (Bialystok et al., 2005, 2014; Hilchey and Klein, 2011). Interestingly, the only study in the review by Paap et al. (2016) that tested task-switching in children reports better switching abilities for three groups (Chinese-English, French-English, Spanish-English) of bilingual children as compared to monolingual children (Barac and Bialystok, 2012).

The number of studies that compare bilingual and monolingual children on a color/shape switching task is limited, but there are studies (e.g., Bialystok, 1999) that use the dimensional change card sorting task (DCCS) (Zelazo, 2006), which is a related but simpler task. In the DCCS task children have to sort cards that show objects in different colors, first according to one dimension (e.g., color), and subsequently according to the other (e.g., shape). In contrast to color/shape switching tasks, the DCCS typically does not include a block in which both sorting rules are mixed, which makes it impossible to derive switching costs and mixing costs. In the DCCS, the ability of children to switch between rules is usually measured by the accuracy scores of the post-switch block, but some computerized versions of the task also measure response times. Whereas most 3-year-olds preserve the first sorting rule when instructed to sort according to a new rule, by the age of 5 most children are able to switch to the new sorting rule without error (Zelazo, 2006).

Bilingual children from different age groups have been found to perform more accurately in the post-switch phase of the card sorting task than monolingual children (Bialystok, 1999 for 3–4 and 5–6 year-olds; Bialystok and Martin, 2004 for three studies with 4–5 year-olds), but some studies report equal performance of bilingual and monolingual children (Yang and Lust, 2004; Gathercole et al., 2014 for accuracy) or even cases where monolingual children outperformed the bilingual groups (Gathercole et al., 2014 for response times). The similar performance of bilingual and monolingual children (mean age: 4.8) in the study by Yang and Lust (2004) may have been caused by ceiling effects for accuracy scores in a post-switch phase. Merely comparing accuracy scores of a post-switch phase might thus not be sufficiently sensitive in groups of children that are already able to switch to a new sorting rule. In such cases, a more complex switching task, such as the cued color/shape switching tasks that has often been used in studies with (young) adults (e.g., Prior and MacWhinney, 2010), is needed. Next to accuracy scores and response times, such a task allows for the calculation of switch and mixing costs. To the best of our knowledge, only Barac and Bialystok (2012) have reported switching costs and mixing costs in a study with bilingual children. Our study will therefore not only compare bilingual and monolingual children for accuracy and response times on task switching but also include switching costs and mixing costs as additional measures of domain-general cognitive control and thus expand our understanding of the effect of (early) bilingualism on cognitive control.

The hypothesis that bilingual language use draws on domain-general control mechanisms has also been tested in studies that looked for relationships between measures of bilingual language control and cognitive control. This line of research focuses on within-group analyses instead of between-group analyses. To test the relation between language control and cognitive control, studies have used different approaches, with diverging results. A number of studies provide evidence for a relationship between bilingual language control and performance on tasks tapping into general cognitive control measures, such as the Flanker task (Festman and Münte, 2012), the Go/NoGo task (Rodriguez-Fornells et al., 2005) and task switching tasks (Prior and Gollan, 2011, 2013; Declerck et al., 2017). Neuroimaging studies moreover suggest that brain areas known to be related to cognitive control are also active during bilingual language use (Abutalebi and Green, 2008; Guo et al., 2011; Luk et al., 2012; Abutalebi et al., 2013; Weissberger et al., 2015), suggesting that there is overlap between mechanisms of bilingual language control and general cognitive control. However, several studies did not find relationships between language switching and tasks of general cognitive control, such task-switching tasks (e.g., Calabria et al., 2011, 2015; Branzi et al., 2016), a flanker task (Declerck et al., 2019) or a Simon task (Jylkkä et al., 2018). Other evidence suggests that the frequency of language switching in real life affects performance on domain-general cognitive measures. Bilinguals who frequently switch between their languages were found to have better interference control (Verreyt et al., 2016) and better cognitive flexibility (Barbu et al., 2018) than bilinguals who switch less frequently.

A possible explanation for the absence of a relationship between language control and cognitive control in many behavioral studies is that these studies tested adults who have been functioning in bilingual settings for many years. Especially for bilingual language use in situations where code-switching is very common and bilinguals use words from both languages without paying attention to the target language, demands on language control mechanisms are likely to be smaller than in situations where one of the languages has to be (partly) inhibited (Green and Abutalebi, 2013), but also language switching in general may be more automatized in experienced bilingual adults and draw less on general cognitive control mechanisms than in bilinguals who have fewer years of bilingual experience, such as bilingual children.

To date, one study has investigated a potential interplay between bilingual language control and general cognitive control in bilingual children. In a recent study with 5- to 7-year-old Spanish-English bilingual children, Gross and Kaushanskaya (2016) tested to what extent children’s performance on a cued color/shape switching task could predict their performance on a cued language switching task. They found that accuracy in nonverbal task switching predicted both naming speed and the number of cross-language intrusion errors (responses given in the non-target language) on cued language switching, indicating that children with better cognitive control were faster and made fewer errors during language switching than children with less developed cognitive control abilities. Whereas task switching accuracy predicted language intrusion errors in both languages, the relationship between task switching and naming speed was only found for the children’s non-dominant language, which – according to the authors – may be caused by the stronger inhibition of the dominant language. Moreover, naming speed on task switching predicted naming speed on language switching. However, similar to studies with adult bilinguals (Calabria et al., 2011, 2015; Branzi et al., 2016; Gross and Kaushanskaya, 2016) did not find any correlations with regard to switching and mixing costs between the two tasks.

The aim of present study was to obtain a better understanding of the interplay between bilingual language control and domain-general cognitive control in bilingual children by comparing nonverbal task switching across bilingual and monolingual children and investigate within the bilinguals, relations between language switching and nonverbal task switching. Specifically, we investigate whether bilingual advantages in nonverbal task switching previously found in one study (Barac and Bialystok, 2012) can be replicated. In addition, we expected that within the bilingual group, language switching abilities would be positively related to nonverbal task switching abilities. This association is possible between accuracy scores and response times from both tasks, as well as switching and mixing costs from both tasks. Investigating this association allows us to test the underlying assumption that bilingual language use is related to cognitive control and a possible consequence, namely that bilinguals have enhanced cognitive control. In so doing, we combine two lines of research that are conceptually closely related, but have been seldomly combined in empirical research.

The bilingual sample in the present study consisted of Turkish-Dutch bilinguals. Children from Turkish-speaking families in the Netherlands are particularly suitable for studying relationships between bilingual language control and cognitive control. In their home environment speakers commonly use both languages, whereas the schools of these children are strictly single-language environments where only Dutch is used. This means that the children frequently find themselves in communicative situations that require a high amount of bilingual language control (Green and Abutalebi, 2013). In previous research, it was moreover found that Turkish-Dutch 5- and 6-year old children showed cognitive benefits in working memory tasks, if socioeconomic status (SES) and language proficiency were statistically controlled (Blom et al., 2014). A similar impact of SES and verbal ability, but with respect to inhibition tasks, was found in research with Spanish-English bilinguals who were 6 years old, on average (Carlson and Meltzoff, 2008). In line with previous studies that provide evidence for better nonverbal switching abilities in bilingual children (Bialystok, 1999; Bialystok and Martin, 2004; Barac and Bialystok, 2012), we also expected better task switching performance of the bilingual Turkish-Dutch children compared to monolingual Dutch children. Based on previous research on working memory and inhibition (Carlson and Meltzoff, 2008; Blom et al., 2014), we expected that bilinguals’ enhanced task switching would surface if SES and verbal ability are controlled.

## Materials and Methods

### Participants

The study included 54 children divided into two groups: 27 Turkish-Dutch bilinguals and 27 Dutch monolinguals. Children were regarded monolingual if Dutch was the only language spoken in the family. For a child to be assigned to the bilingual group at least one of the child’s parents had to speak Turkish in the home environment. At the time of testing, all children were between 5 and 8 years old (mean age = 7.5). We matched the two groups on age and nonverbal intelligence scores (NVIQ) (Table 1). Non-verbal intelligence was measured with the short version of the Wechsler Nonverbal-NL (Wechsler and Naglieri, 2008). There was no significant age difference between the groups (*F*(1,54) = 0.22, *p >* 0.05, η_p_^2^ = 0.004) and no significant difference between the groups in NVIQ (*F*(1,54) = 0.006, *p* > 0.05, η_p_^2^ < 0.001). We furthermore aimed to create groups that were comparable on socioeconomic status (SES) and Dutch receptive vocabulary outcomes. SES was indexed by the average educational level of both parents of the child, based on the Questionnaire for Parents of Bilingual Children (PaBiQ; Tuller, 2015). Receptive vocabulary in Dutch was measured with the Peabody Picture Vocabulary Test (PPVT-III-NL; Schlichting, 2005). However, despite our efforts, SES did differ significantly across the groups (*F*(1,54) = 7.1, *p* = 0.01, η_p_^2^ = 0.12), reflecting slightly lower socioeconomic positions of Turkish families in the Netherlands as compared to native Dutch (monolingual) families. There was also a significant difference between the two groups for Dutch receptive vocabulary scores: *F*(1,54) = 16.7, *p* < 0.001, η_p_^2^ = 0.24, indicating higher scores for the monolingual children than for the bilingual children, as has been found in previous studies (Bialystok et al., 2010).

**TABLE 1:** Average age in months, nonverbal IQ scores, socioeconomic status and Dutch receptive vocabulary per group.

	N	Age (SD)	NVIQ (SD)	SES (SD)	PPVT (SD)
Monolinguals	27	91.5 (6.0)	101.9 (12.3)	6.1 (2.1)	105.6 (10.1)
Bilinguals	27	90.5 (9.4)	102.1 (13.2)	4.6 (2.0)	91.4 (14.9)

*NVIQ, nonverbal intelligence standardized score; SES, socioeconomic status, average educational level of both parents measured on a nine-point scale; PPVT, Peabody Picture Vocabulary Test, receptive Dutch vocabulary score converted to standardized age-corrected normative scores (M = 100, SD = 15).*

Table 2 gives an overview of the proportions of language use (Dutch, Turkish) in the home environment of the bilingual children and language proficiency in both languages. Information on language use at home was collected with the PaBiQ (Tuller, 2015), Dutch language proficiency scores were based on the Dutch PPVT, and Turkish language proficiency scores were based on a Turkish translation of the PPVT (see section “Materials and Methods” for more information). The receptive vocabulary scores in Table 2 show the percentages of correct items.

**TABLE 2:** Proportion of language use (percentage of time, with SD and range) of the bilingual children in the home environment and % accuracy for receptive vocabulary in the two languages (with SD and range).

	Language use at home in % (SD)	Range	Receptive vocabulary in % correct (SD)	Range
Dutch	41.6 (10.8)	21.4–66.7	50.6 (14.0)	20.0–72.5
Turkish	58.4 (10.8)	33.3–78.6	57.8 (12.2)	27.5–77.5

On average, Turkish was used more often than Dutch (*t*(22) = 3.7, *p* = 0.001). According to the parental questionnaire data, 70% of the families used Turkish more frequently than Dutch at home, 17% used Dutch more frequently than Turkish and 13% used the two languages equally often. In addition, the majority of parents (87%) reported that they mixed the two languages in the home environment. On average, receptive vocabulary scores are a bit higher for Turkish than for Dutch (*t*(26) = 2.5, *p* = 0.02), but the ranges and standard deviations show that there is much variation within this group. It is important to note that all of these children had started elementary school, where Dutch is the only language of instruction, at age 4. Thus, whereas the children are in a dual-language situation at home, they are in a single-language situation at school.

### Background Information

#### Language Use at Home

Information on bilingual language use at home was gathered by using a parental questionnaire based on the PaBiQ (Tuller, 2015). Turkish-Dutch bilingual assistants administered the questionnaire during a telephone interview with one of the child’s parents. Dutch language use was measured as frequency with which a child was addressed in Dutch in the home environment. This information was collected for the mother, father, other caregivers and siblings on a five-point scale ranging from 0 = *never* to 4 = *always*. The same measure was applied for use of Turkish. Information regarding other caregivers was only included when these individuals were present in the home environment at least several times per week. The frequency of language use in the home environment was calculated for each language.

#### Language Proficiency

Receptive vocabulary size was assessed by the Dutch Peabody Picture Vocabulary Task (PPVT-III-NL; Schlichting, 2005). The PPVT is a standardized receptive vocabulary test designed for the age range from 2 years and 3 months up to 90 years and contains 204 items divided over 17 sets. Each set consists of 12 items and the level of difficulty increases throughout the sets. In this task, children heard a stimulus word and had to choose the correct referent out of four pictures. The PPVT-III-NL was administered and scored according to the official guidelines: the starting set was determined by a child’s age and the task was terminated after a child produced nine or more errors within one set. Raw scores were converted to standardized scores based on age-corrected norms. These standardized scores were used for the matching of bilingual and monolingual children. For the bilingual children, we also administered a Turkish version, which was a translation of the Dutch task for which permission was obtained from the publisher (Blom, 2019). The translation of the task was done by a bilingual speaker of Turkish and Dutch. Turkish items that were cognates or – according to the bilingual translator – not comparable to the Dutch item with regard to difficulty were deleted, which resulted in a task with 8 items per set instead of 12. To compare vocabulary skills in both languages, we calculated the percentage of correct answers for all the items that were used in both the Dutch and the Turkish versions of the task as presented in Table 2.

### Switching Tasks

#### Language Switching

Language switching was measured in the bilingual group with a cued picture naming task that was developed with the software package E-Prime 2.0 Standard (Psychology Software Tools, Pittsburgh, PA, United States). The task included 32 colored pictures of objects, selected from a picture database (Rossion and Pourtois, 2004). Pictures were chosen to refer to highly frequent concrete Dutch nouns (based on SUBTLEX-NL; Keuleers et al., 2010) that were all included in a list of words that children in the Netherlands are expected to be familiar with in kindergarten (Basiswoordenlijst Amsterdamse Kleuters (BAK); Mulder et al., 2009). This was done so that the task would test the children’s ability to rapidly access words and not their knowledge of words. To ensure that the level of difficulty of naming these words in Turkish was comparable to naming these words in Dutch, only pictures that native speakers rated as “very easy” were included. None of the words for pictures in the task were cognates between Dutch and Turkish.

All items in the task were divided into lists by first creating pairs of words that were from the same semantic category, e.g., “animal,” and were comparable with regard to word frequency and word length, and then assigned the two words of each pair to different lists. For example, the word “cat” in list A would be matched with the word “dog” in list B. This resulted in two comparable word lists with 14 different items each for each language (see Appendix 1).

Each of the single language blocks consisted of two practice trials and 28 test trials. Children either started with Dutch or Turkish. The order of the two languages was counterbalanced among the participants. The order of the two word lists remained the same for the single language blocks so that children who started with Dutch named word list A in Dutch followed by word list B in Turkish and children who started with Turkish named list A in Turkish and list B in Dutch. The mixed language block always followed the two single language blocks and consisted of four practice trials and 56 test trials which consisted of 28 trials per language. The order of the trials was fixed. The target language changed every 2 to 5 trials. The target language stayed the same as in the previous trials for 75% of the trials and changed to the other language for 25% of the trials.

The language switching task was presented on a 15-inch laptop screen. The two single language blocks introduced two different interlocutors, a cartoon face of girl and a cartoon face of a boy (see Appendix 2). The girl was introduced as a monolingual speaker of Dutch and gave instructions for the Dutch language block. The face of the boy was introduced as a monolingual speaker of Turkish and explains the Turkish single language block. The instructions for the mixed language condition were explained both in Dutch (by the girl) and in Turkish (by the boy).

The purpose of introducing the two faces was to cue the language of the test condition during the task. The girl’s face served as language cue for Dutch and the boy’s face was the language cue for naming in Turkish. The children were familiarized with the language cues during the single language blocks, where they were the same for all trials of a block. In the mixed language condition children had to respond either in Dutch or in Turkish, depending on the cue that was located above the target item. By introducing two interlocutors that differed in the language in which they had to be addressed, the task was assumed to better resemble a real-life mixed language situation than when arbitrary cues, e.g., colors, would have been used (Peeters and Dijkstra, 2018). In each language, the cue was presented for 650 ms, followed by a fixation cross for 350 ms, then a blank screen for 150 ms, and then the target picture. The target picture remained on the screen until a response was given. After the child’s response the test assistant clicked the mouse to proceed to the following item. This was done to prevent data loss due to the child’s inattention. Test assistants were instructed to only click to the next item if the child was still paying attention and was not distracted. This procedure was practiced with all test assistants prior to testing and test assistants were instructed to keep up a steady pace to minimalize variability in response-to-cue interval. The cue remained on the screen until the end of the trial. There was a time limit of 7,000 ms for the child to respond.

Children’s spoken responses were picked up by a microphone connected to a PST serial response box with a voice key function. Responses were also recorded via an external USB microphone for offline scoring of accuracy.

#### Nonverbal Task-Switching

The color/shape switching task was designed in E-Prime 2.0 Standard (Psychology Software Tools, Pittsburgh, PA, United States) and had a largely similar design as the language switching task. It consisted of two single task blocks and a cued task switching block. Children were presented blue or orange triangles or squares and for each trial they had to respond to either the color (blue vs. orange) or the shape (triangle vs. square) of the target. Before each single task block a cartoon face showing “Mr. Color” or “Mr. Shape” (see Appendix 2) gave instructions on the task rules. The two faces also served as task cues for the switching block. By introducing the cues already in the single task blocks children were able to familiarize themselves with the cues. During all trials children saw a blue square in the left bottom corner and an orange triangle in the right bottom corner. For each trial children had to respond by pressing one of two fixed buttons on the far left and far right sides of the keyboard. When responding to color the left button was for *blue* and the right button for *orange*. When responding to shape the left button was for *square* and the right button was for *triangle*. This was in line with the symbols they saw in the bottom corners of the screen and was additionally indicated by stickers on the corresponding keys. Other details regarding the design of the task, such as number of trials and length of duration of cues and stimuli was exactly the same as in the language switching task.

### Data Preparation

#### Language Switching

For each language, accuracy scores were calculated as the percentage of correct trials during the mixed language block. Scoring was done by trained assistants using the audio recordings. For calculations of mean response times (RTs), only accurate trials were used. Response latencies were measured as the interval between picture presentation and onset of the target response, disregarding all audible noise or filled pauses preceding the target response. Trials in which a child said something else prior to the target word (e.g., “I know this one, tree”) were excluded. All RTs smaller than 200 ms were excluded and all RTs smaller than 500 ms were checked and measured manually to determine e.g., if the voice key had been triggered accidentally by other sounds, such as background noise. For each child we computed means and standard deviations per language and trial type (repeat vs. switch trial). The first trial of the mixed language block was excluded as this is neither a repeat nor a switch trial. All trials that were 3 standard deviations above the mean were excluded. Together with trials yielding incorrect responses, this led to the exclusion of 9.1% of the data. Per language, we calculated two types of costs, switching costs and mixing costs. Switching costs were calculated per child by subtracting the mean response time on repeat trials from the mean response time on switch trials. Mixing costs were calculated by subtracting the mean response time on trials from the single language block from the mean response time on repeat trials in the mixed language block.

#### Nonverbal Task-Switching

Paired samples *t*-tests showed that there were no significant differences between the single task conditions for color and shape, neither for accuracy scores (*t*(26) = 1.04, *p* = 0.31), nor for response times (*t*(25) = 1.04, *p* = 0.31). Therefore, color and shape trials were pooled for analyses of the task-switching block, which resulted in four measures for task-switching: overall accuracy during the switching block, mean response time during the switching block, switching costs (difference in response times between switch and repeat trials during the switching block), and mixing costs (difference in response times between the repeat trials of the switching block and the average response times of the two single task blocks). Mean response times were calculated only for accurate trials and trials with response times >200 ms. Trials with response times that were above three standard deviations above a child’s mean were not included. Together with excluding incorrect responses, this led to the exclusion of 15.1% of the data.

### Procedures

The research was screened by the Standing Ethical Assessment Committee of the Faculty of Social and Behavioral Sciences at Utrecht University. Criteria were met and further verification was not deemed necessary. Parents of participating children signed an informed consent. Children were tested individually in a quiet room at their schools. The tests were administered by trained assistants following a standardized protocol. The tasks used for this study were part of a larger test battery divided into two test sessions, with 1 week in between the two sessions. In the bilingual sample, the language switching task was part of the first test session whereas the nonverbal task switching was part of the second test session. Monolinguals did not engage in language switching. They completed the nonverbal switching task in the first test session. The parental questionnaire was administered during a telephone interview with one of the child’s parents. The interview was conducted by bilingual assistants who were proficient in both Dutch and the heritage language of the child, and could therefore be carried out in the preferred language of the parent. Per language the percentage of language use in the home environment was calculated. SES was measured by level of education on a nine-point scale for both the mother and the father of the child. Averages of both parents were calculated and used for the analyses as a covariate.

## Results

### Comparing Bilingual and Monolingual Children on Nonverbal Task-Switching

Table 3 shows the accuracy, response times, switching costs and mixing costs in the bilingual and monolingual samples in the mixed task condition.

**TABLE 3:** Average accuracy, response times, switching costs, and mixing costs in nonverbal task-switching for the monolingual and bilingual group (mixed task condition).

	N	Accuracy in % (SD)	RTs (SD)	Switching costs (SD)	Mixing costs (SD)
Monolinguals	27	82.3 (12.5)	1247.0 (405.4)	137.7 (202.7)	338.2 (218.8)
Bilinguals	27	78.1 (10.6)	1273.3 (345.2)	265.4 (396.2)	471.2 (280.2)

*RTs, response times.*

Before comparing the groups, we inspected correlations to determine the strength of interrelationships between the four dependent variables (see Table 4). Accuracy showed a positive correlation with switching costs, indicating that children who made fewer errors needed relatively more time between switch and repeat trials than children who made more errors, pointing to a trade-off effect. Mixing costs showed a positive correlation with overall response times, demonstrating that children who needed relatively much time to respond to repeat trials in the mixing condition, were also overall relatively slow in responding in the mixing condition. There was no overall speed-accuracy trade-off.

**TABLE 4:** Correlations between accuracy, response times, switching costs, and mixing costs in nonverbal task-switching (both groups collapsed; mixed task condition).

	Accuracy	RTs	Switching costs
Accuracy			
RTs	0.05		
Switching costs	0.29*	0.10	
Mixing costs	−0.05	0.78**	0.12

RTs, response times. *p < 0.05, **p < 0.01.

We conducted Multivariate Analysis of Variance (MANOVA) analyses. A MANOVA is a more powerful test that is able to identify smaller effects than a regular ANOVA by taking into account correlations between different dependent variables. Two MANOVA’s were conducted that combined those outcome measures that were correlated: (1) accuracy and switching costs, and (2) RTs and mixing costs. Each MANOVA was followed by a MANCOVA in which SES and Dutch receptive vocabulary were included as covariates. The first MANOVA returned a non-significant effect for accuracy and switching costs (*F*(2,51) = 2.96, *p* = 0.06, η_p_^2^ = 0.10); a trend suggested that the bilinguals had lower accuracy and larger switching costs. The follow-up MANCOVA returned a clearly non-significant effect (*F*(2,49) = 2.96, *p* = 0.51, η_p_^2^ = 0.03). The second MANOVA showed a significant effect for RTs and mixing costs (*F*(2,50) = 3.77, *p* = 0.03, η_p_^2^ = 0.13), indicating that the bilinguals had larger RTs and higher mixing costs. The follow-up MANCOVA returned a non-significant effect (*F*(2,48) = 1.44, *p* = 0.25, η_p_^2^ = 0.06). In summary, the results show that any differences between monolinguals and bilinguals are related to differences in SES and knowledge of Dutch. When these factors are controlled, there are no differences between monolinguals and bilinguals on nonverbal task-switching.

### Language Switching and Nonverbal Task-Switching in Bilingual Children

To test whether bilingual language control and domain-general cognitive control are related in the bilingual group we computed Pearson correlations between the dependent measures drawn from the nonverbal switching task and the language switching task. Accuracy scores from the language switching task were at ceiling (mean >85%) and therefore not included. There was a marginally significant moderate correlation between accuracy on nonverbal task-switching and response times for Dutch trials during language switching, indicating that higher accuracy at nonverbal task switching is related to faster response times during language switching, *r*(25) = −0.39, *p* = 0.06. Accuracy on nonverbal task-switching was not related to response times of Turkish trials during language switching, *r*(25) = −0.11, *p* = 0.61. Analyses of the correlations between the mean response times of the two tasks showed that children’s response times during nonverbal task switching showed a significant, positive correlation with children’s response times during language switching and that this was the case for Dutch trials, *r*(25) = 0.45, *p* = 0.02, as well as Turkish trials, *r*(25) = 0.50, *p* = 0.011. Switching costs (Dutch: *r*(25) = 0.11, *p* = 0.59; Turkish: *r*(25) = 0.04, *p* = 0.86) and mixing costs (Dutch: *r*(24) = −0.01, *p* = 0.96; Turkish: *r*(24) = 0.21, *p* = 0.33) of the two tasks were unrelated.

To ensure that the correlations between response times on the two tasks were not affected by confounding factors, we ran four separate partial correlations with age, NVIQ, SES and vocabulary in Dutch as control variables (Table 5). Compared to the correlations where these factors were not controlled for, most of the correlation coefficients either increased in size or stayed similar. All partial correlations between response times on language switching and nonverbal task-switching were significant, indicating that the relationship between response times on the two switching tasks cannot be attributed to individual differences between children in age, NVIQ, SES or vocabulary scores.

**TABLE 5:** Partial Pearson’s correlations between response times of nonverbal task-switching (mixed task block) and language switching (mixed language block) controlling for age, nonverbal IQ, socioeconomic status and proficiency in Dutch.

	Control variable	RTs Dutch	RTs Turkish
RTs	Age	*r* (22) = 0.43, *p* = 0.04	*r* (22) = 0.50, *p* = 0.01
nonverbal	NVIQ	*r* (22) = 0.46, *p* = 0.02	*r* (22) = 0.50, *p* = 0.01
task-	SES	*r* (22) = 0.49, *p* = 0.02	*r*(22) = 0.55, *p* = 0.005
switching	PPVT	*r* (22) = 0.48, *p* = 0.02	*r* (22) = 0.51, *p* = 0.01

*RTs, response times; NVIQ, nonverbal intelligence; SES, socioeconomic status; PPVT, Dutch receptive vocabulary measured with the Peabody Picture Vocabulary Test.*

Additionally, we computed Pearson correlations between the response times from the single language blocks of the language switching test (Dutch, Turkish) and the single task blocks (color, shape) from the nonverbal task-switching test to make sure that the relationship between response times in the mixed blocks of the language switching test and the nonverbal task-switching test did not merely reflect individual differences in task speed in general. One correlation may suggest a trend (RTshape-RTTurkish: *r*(24) = 0.37, *p* = 0.08), but most of the correlations were far from significant (RTcolor-RTDutch: *r*(25) = 0.11, *p* = 0.60; RTcolor-RT Turkish: *r*(25) = −0.01, *p* = 0.97; RTshape-RTDutch: *r*(24) = 0.13, *p* = 0.56). It is thus unlikely that the correlations between response times in the switching blocks of the two tests simply reflect associations between performance speed on the two tests.

## Discussion and Conclusion

The current study investigated if Turkish-Dutch bilingual children outperform their monolingual peers on nonverbal switching, and if language switching and nonverbal switching are related to each other within the sample of Turkish-Dutch bilingual children.

Starting with the second relationship, we found that response times on language switching and response times on nonverbal task-switching were significantly related: children who are better at switching between Turkish and Dutch are also better at switching in a nonverbal task in which they have to switch between a shape and color sorting rule. These results are in line with a recent similar study that tested cued task-switching in Spanish-English bilingual children (Gross and Kaushanskaya, 2016). As Gross and Kaushanskaya (2016) mention, it is possible that this association reflects similar speed demands of the two tasks. However, since the relationship in our study only emerged for the response times during mixed language/nonverbal task blocks and not during single language/nonverbal task blocks, we conclude that this finding provides evidence for shared domain-general control mechanisms that are utilized for switching between languages and between nonverbal tasks. This relationship was robust and not confounded by factors such as age, nonverbal intelligence, socioeconomic status or language proficiency.

The data showed a trend that accuracy on nonverbal task-switching was related to response times for the Dutch trials during language switching but not to response times for the Turkish trials. Gross and Kaushanskaya (2016) only found this relationship for the non-dominant language of the children, irrespective of whether this was English or Spanish. Because of different patterns in bilingual language use in our participants, it was not possible for us to make a distinction between the children’s dominant versus non-dominant language rather than distinguishing between Dutch and Turkish. We can therefore neither confirm nor refute the idea that naming pictures in the non-dominant language (as opposed to the dominant language) draws on domain-general cognitive control mechanisms. Similar to previous research, both on bilingual children (Gross and Kaushanskaya, 2016) and adults (Calabria et al., 2011, 2015; Branzi et al., 2016), our study did not find direct relationships between the processing costs (switching/mixing costs) caused by language switching and nonverbal task-switching, although there are also studies that report relationships between language switching and task switching with regard to these measures (Declerck et al., 2017; Timmer et al., 2018).

The assumption that bilingual language control draws on domain-general cognitive control has also been used to explain why bilingual children outperform their monolingual peers on tasks tapping into cognitive control (Bialystok, 1999; Bialystok and Martin, 2004). However, despite significant relations between language switching and nonverbal task-switching in the bilingual group, our results do not provide any evidence for better nonverbal task-switching abilities in the bilingual group as compared to a monolingual control group, neither based on accuracy or on response times, switching or mixing costs. This is different from some previous studies with children using a dimensional change card sort task (DCCS) (Bialystok and Martin, 2004) or a very similar color/shape switching task (Barac and Bialystok, 2012).

Unlike Barac and Bialystok (2012), we could not match the two language groups on socioeconomic status. Moreover, whereas two of the bilingual groups in the study of Barac and Bialystok (2012) show slightly lower English vocabulary scores than the monolingual children and one bilingual group, the difference in Dutch vocabulary scores between the two groups in our study was considerably larger. However, even when socioeconomic status and verbal ability were statistically controlled, the bilingual children did not outperform their monolingual peers on nonverbal task-switching. In matching, we focused on a number of factors that are most likely to differ across the bilinguals and monolinguals in our study. In addition, we co-varied those factors that could not be matched in order to exclude confounding variables. Unfortunately, we were unable to match the groups on all factors that have been shown to impact cognitive control, e.g., playing a musical instrument (Musacchia et al., 2007; Moradzadeh et al., 2014) or playing computer games (Merzenich et al., 1996; but see also Unsworth et al., 2015). It is possible that factors like these are unequally distributed across the two groups, and create a confound. In addition to confounding variables, it is important to consider whether our study had sufficient power to detect a difference between the two groups. The samples in our study were similar in size to those of Barac and Bialystok (2012) who did find a significant effect using a similar task. A power calculation based on the reported effect size in this this previous study suggests that our study was not underpowered. However, the task used in our study had fewer trials than the task used in the study by Barac and Bialystok (2012). The absence of an effect ties in with other research that failed to find an effect of bilingualism on other cognitive control tasks (Duñabeitia et al., 2014). It confirms the conclusion that cognitive effects lack stability and robustness (Paap et al., 2015), and may depend on specific properties of the sample, such as age (Bosma et al., 2017).

In conclusion, as the relationship between bilingual language control and cognitive control is the underlying assumption for potentially enhanced cognitive control in bilingual as opposed to monolingual speakers, the current study combined both types of study. The results demonstrated that bilingual children with better nonverbal cognitive control have better language control, which is consistent with the hypothesis that domain-general cognitive resources are utilized for language switching (Green, 1998; Green and Abutalebi, 2013). Importantly, this relationship does not necessarily entail a cognitive training effect in bilinguals, at least not to the extent that the bilingual children outperform their monolingual peers on a task tapping into cognitive control. In fact, without controlling for differences in socioeconomic status and Dutch receptive vocabulary, the monolinguals outperformed the bilinguals on cognitive control. When both factors were controlled, the monolingual advantage disappeared. These outcomes have important implications for the debate on bilingual children’s cognitive advantages, as they demonstrate that finding no cognitive advantages cannot be taken as evidence for the absence of a relation between language control and cognitive control. Moreover, the results suggest that bilingual-monolingual comparisons involve factors that exert greater influence on cognitive control than frequent practice in language switching does, and that such (confounding) factors may even lead to observing monolingual instead of bilingual cognitive control advantages.

## Data Availability Statement

The datasets generated for this study are available on request to the corresponding author.

## Ethics Statement

The studies involving human participants were reviewed and approved by Ethical Assessment Committee of the Faculty of Social and Behavioral Sciences at Utrecht University. Written informed consent to participate in this study was provided by the participants’ legal guardian/next of kin.

## Author Contributions

MT, PL, FW, and EB contributed to the conceptualisation and design of the study. MT analyzed the data and wrote the first draft of the manuscript. All authors contributed to revisions, read and approved the submitted version.

## Conflict of Interest

The authors declare that the research was conducted in the absence of any commercial or financial relationships that could be construed as a potential conflict of interest.

## Figures and Tables

**FIGURE 1:**
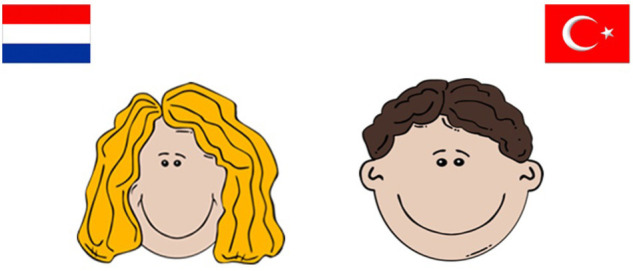
Introduction of cues on language switching task.

**FIGURE 2:**
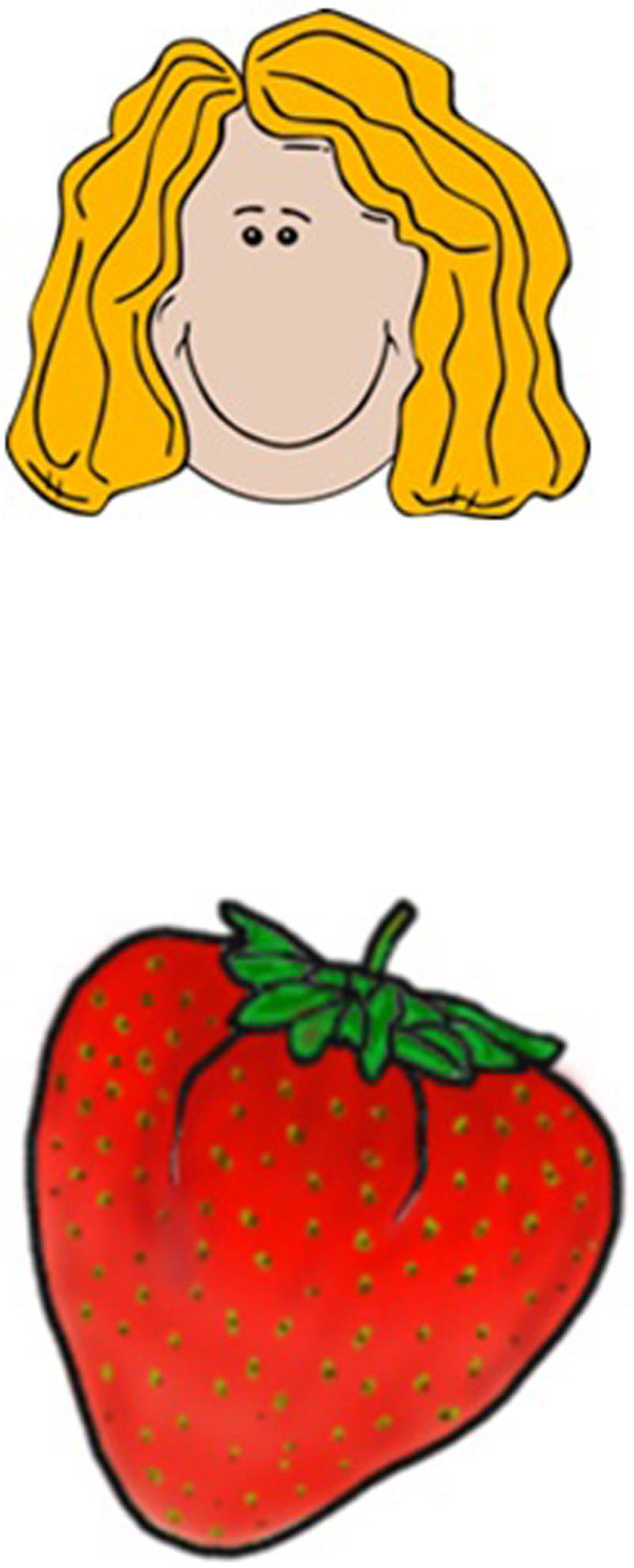
Example of a Dutch language trial on language switching task.

**FIGURE 3:**
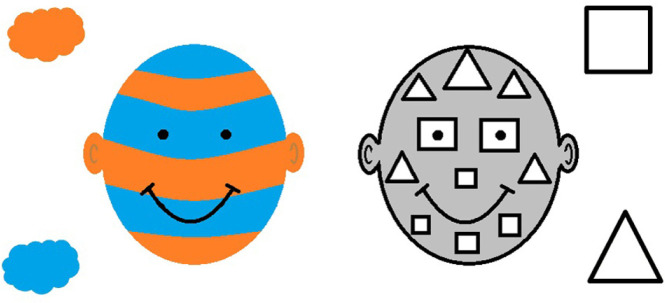
Introduction of cues on the nonverbal switching task.

**FIGURE 4:**
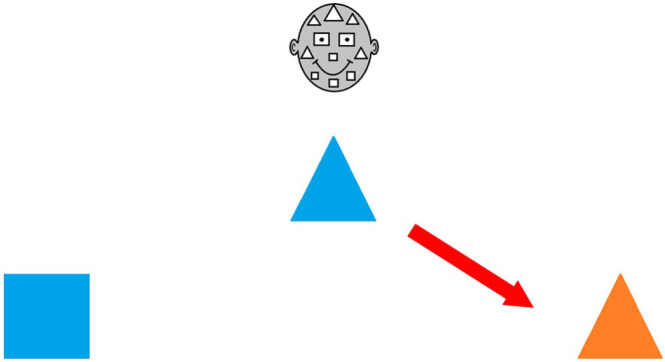
Example of a shape trial (the arrow only appeared during the instructions).

**FIGURE 5:**
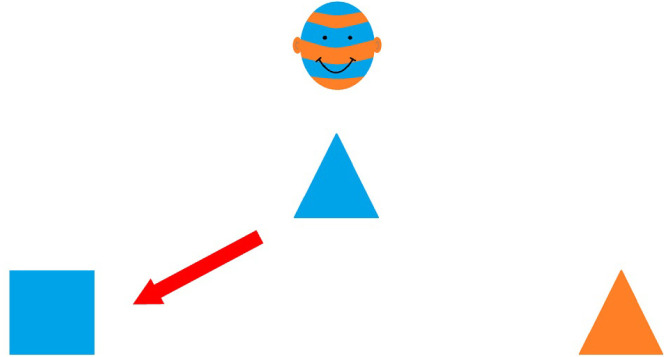
Example a color trial (the arrow only appeared during the instructions).

## Appendix 1 List of Picture Names from the Language Switching Task

**Table:**

Dutch	Turkish	English translation
vis	balık	fish
oog	göz	eye
hart	kalp	heart
appel	elma	apple
deur	kapı	door
mes	bıçak	knife
auto	araba	car
vogel	kus̨	bird
sleutel	anahtar	key
oor	kulak	ear
varken	domuz	pig
bank	koltuk	couch
konijn	tavs̨an	rabbit
boom	ağaç	tree
kip	tavuk	chicken
neus	burun	nose
ster	yıldız	star
wortel	havuç	carrot
bed	yatak	bed
vork	çatal	fork
fiets	bisiklet	bike
paard	at	horse
schaar	makas	scissors
vinger	parmak	finger
schaap	koyun	sheep
tafel	masa	table
olifant	fil	elephant
bloem	çiçek	flower
